# Phylogenetic assessment within a species complex of a subterranean rodent (*Geomys bursarius*) with conservation implications for isolated subspecies

**DOI:** 10.1093/jhered/esae035

**Published:** 2024-07-10

**Authors:** Nathan Alexander, Alida de Flamingh, Bradley J Cosentino, Robert L Schooley

**Affiliations:** Department of Natural Resources and Environmental Sciences, University of Illinois, Urbana, IL, United States; Carl R. Woese Institute for Genomic Biology, University of Illinois, Urbana, IL, United States; Department of Biology, Hobart and William Smith Colleges, Geneva, NY, United States; Department of Natural Resources and Environmental Sciences, University of Illinois, Urbana, IL, United States

**Keywords:** evolutionary significant unit, *Geomys bursarius illinoensis*, mitogenomics, phylogenetics, plains pocket gopher

## Abstract

Range contraction and expansion from glaciation have led to genetic divergence that may be particularly pronounced in fossorial species with low dispersal. The plains pocket gopher (*Geomys bursarius*) is a fossorial species that ranges widely across North America but has a poorly understood phylogeny. We used mitogenomes (14,996 base pairs) from 56 individuals across seven subspecies, plus two outgroup species, to assess genetic divergence from minimum spanning trees, measure genetic distances, and infer phylogenetic trees using BEAST. We found *G. b. wisconsinensis* was monophyletic with recent divergence. Further assessment is needed for *G. b. major* because it was paraphyletic and exhibited inconsistent groupings with other clades. Importantly, we identified *G. b. illinoensis* as being genetically distinct and monophyletic likely due to a unique colonization event eastward across the Mississippi River. Because *G. b. illinoensis* faces continued pressures from niche reduction and habitat loss, we recommend that *G. b. illinoensis* be considered an evolutionary significant unit warranting conservation actions to promote connectivity and restore suitable habitat. Such conservation efforts should benefit other grassland species including those originating from clades west of the Mississippi River that may also be evolutionary significant units.

## Introduction

Phylogeography is important for understanding the role of historic range expansion and colonization on contemporary genetic structures of taxa and providing context for conservation efforts (Wang 2010; Epps and Keyghobadi 2015; Rissler 2016). Phylogeography and phylogenetics can be particularly useful for clarifying intraspecific genetic variation and intraspecific relationships. By describing the phylogeography of subspecies, we can provide insight into higher levels of taxonomic classification by bridging micro- and macro-evolutionary processes (Avise 2009; Avise et al. 2016; Rissler 2016). Such resolution also is needed to clarify whether isolation has produced evolutionary significant units that may require targeted conservation interventions (Moritz 1994; Mace 2004; Hoelzel 2023).

A major driver of genetic variation is range contraction and expansion in response to glacial ice sheets with the Pleistocene glaciation often creating subspecies or population-level genetic structure (Arbogast and Kenagy 2001; Brant and Ortí 2003). In North America, responses to glaciation have shaped species distributions and genomes, with species expanding northward with the retreating glacial sheets (Hewitt 2000). However, with multiple glaciation events, populations expanding northward were likely periodically isolated, or went extinct and experienced recolonization, creating a complex system for understanding speciation events and intraspecific genetic variation (Russell 1968; Hewitt 2000). As such, species that expanded northward from southern glacial refugia have varying and complicated phylogeographic relationships. Populations in the Midwest region of the United States often show structuring from northward expansions from glacial refugia in Texas and Mexico (Starkey et al. 2003; Du et al. 2019; Ford et al. 2020; Blais et al. 2021). However, striped skunks (*Mephitis mephitis*), American badgers (*Taxidea taxus*), and gray foxes (*Urocyon cinereoargenteus*) in the Midwest demonstrate substantial admixture without strong signals from post-glaciation expansion (Barton and Wisely 2012; Ford et al. 2020; Reding et al. 2021). This variation in post-glaciation genetic signatures may be due to different locomotion and dispersal abilities of taxa under changing climates, with taxa facing unique barriers or having distinct expansion rates (Feder et al. 2009; Schloss et al. 2012).

Subterranean rodents with low dispersal abilities can retain localized genetic structure from historic range expansion after glaciation (Elrod et al. 2000; Alexander et al. 2024). Thus, pocket gophers (Geomyidae) are ideal for understanding phylogeographic patterns within a broader species complex (Avise et al. 1979; Sudman et al. 2006; Chambers et al. 2009). Pocket gophers in the genus *Geomys* range broadly across central North America, from northern Mexico to southern Canada, and from Florida to New Mexico (Sudman et al. 2006; Chambers et al. 2009). Classification of the *Geomys* species complex has a long history (Russell 1968; Hart 1971; Sudman et al. 2006; Genoways et al. 2008; Chambers et al. 2009), with previous work elevating races to subspecies and, in some cases, elevating races and subspecies to species (Bohlin and Zimmerman 1982; Block and Zimmerman 1991; Elrod et al. 2000; Sudman et al. 2006; Genoways et al. 2008). Currently, *Geomys* includes four species groups: *G. bursarius*, *G. breviceps*, *G. personatus*, and *G. pinetis* (Sudman et al. 2006; Chambers et al. 2009). The *G. bursarius* group contains between one and six species (Sudman et al. 2006; Chambers et al. 2009) with the *G. bursarius* species including eight subspecies (*G. b. bursarius*, *G. b. illinoensis*, *G. b. industrius*, *G. b. major*, *G. b. majusculus*¸ *G. b. missouriensis*, *G. b. ozarkensis*, and *G. b. wisconsinensis*) that occur across the Great Plains and part of the Midwest (Connior 2011).

*Geomys bursarius* likely has undergone both adaptive and non-adaptive divergence (Hart 1971) with glacial events leading to isolated populations and multiple range expansions northward (Russell 1968). Intraspecific phylogenetics of *Geomys* remain largely unexplored with resolution limited due to a focus on karyotypes, targeted nuclear gene sequencing, or specific mitochondrial genes (Hart 1971; Smolen and Bickham 1995; Elrod et al. 2000; Sudman et al. 2006; Chambers et al. 2009). The subspecies within *G. bursarius* need further examination as some genetic distances show divergence at levels potentially warranting species-level delimitation (Baker and Bradley 2006; Chambers et al. 2009).

Here, we expand phylogenetic analyses of pocket gophers by focusing on the *G. bursarius* clade with particular attention to resolving the position of *G. b. illinoensis*. Soil properties and rivers can act as barriers to *Geomys* species, subspecies, and races (Baker et al. 1973; Sudman et al. 1987; Genoways et al. 2008; Alexander et al. 2024). In some regions, soil transitions can create contact zones and thus allow hybridization and introgression, further complicating *Geomys* taxonomy (Baker et al. 1973, 1989; Burns et al. 1985; Dowler 1989; Genoways et al. 2008). Whereas soils may introduce contact zones, major waterways like the Mississippi, Missouri, and Illinois Rivers act as barriers across the range of *G. bursarius*, genetically isolating populations (Alexander et al. 2024) likely due to the poor swimming ability of gophers (Kennerly 1963).

The Illinois River acts as a northern boundary for *G. b. illinoensis*, whereas historic expansion across the Mississippi River followed by genetic isolation offers an excellent opportunity to understand northward range expansion post-glaciation (Smith 1957; Connior 2011; Alexander et al. 2024). Dispersal events across the Mississippi River should be rare, which may promote speciation of *G. b. illinoensis*. As contemporary fragmentation and niche reduction of *G. b. illinoensis* continues (Alexander et al. 2022) due to extensive loss of grassland habitat and ongoing agricultural intensification (Ramankutty and Foley 1999; Augustine et al. 2021), it is increasingly critical to understand the genetic distinctness of *G. b. illinoensis* because it is facing contemporary threats due to landscape change and fragmentation.

We use complete mitochondrial genomes (mitogenomes) to clarify the relationships of *G. bursarius* subspecies across the central United States, specifically determining if clades such as *G. b. illinoensis* are monophyletic and meet recommendations for being a unique genetic population of conservation concern (Moritz 1994; Mace 2004). Through using well-established methods for phylogenetic tree inferences, we account for high variation in the mitogenomes and produce holistic insights regarding the phylogeny of *G. bursarius*. Specifically, we 1) generated minimum and median spanning networks to interpret haplotype structure, 2) estimated genetic distances between individuals, and 3) inferred phylogenetic trees to determine branch support and establish if subspecies were monophyletic to determine phylogenetic structure of *G. bursarius* and assess genetic distinctiveness of subspecies.

## Methods

### Tissue samples and DNA extraction

We identified *G. bursarius* samples analyzed by Alexander et al. (2024) that had high DNA content based on microsatellite amplification to increase the likelihood of reconstructing complete mitogenomes. These samples included 41 toes from museum specimens from the Illinois Natural History Survey collected between 1934 and 1985, and 12 tissue samples from *G. bursarius illinoensis* live-trapped in 2018 to 2019, following appropriate guidelines (UIUC IACUC #17190; Sikes and The Animal Use and Care Committee of the American Society of Mammalogists 2016). We sampled seven *G. bursarius* subspecies from across the range, excluding only *G. b. ozarkensis*. All museum samples were assigned to subspecies by respective collectors except for five samples from Wisconsin (Supplementary Materials 1). We assigned these Wisconsin samples as *G. b. wisconsinensis* based on the range map (Connior 2011). We included three samples from other *Geomys* species (one *G. breviceps dutcheri* and two *Geomys jugossicularis*) as outgroups. *Geomys jugossicularis* was previously considered within the *G. bursarius* clade but was determined to be an independent species (Sudman et al. 2006; Chambers et al. 2009). For full DNA extraction methods see Alexander et al. (2024). Arbor Biosciences (arborbiosci.com; Ann Arbor, MI, USA) completed library preparation, mtDNA-bait enrichment and capture, and genome sequencing. Arbor Sciences created customized mtDNA-baits using a myBaits kit to enrich *Geomys* mtDNA prior to sequencing. Baits were customized using a *G. personatus* reference mitogenome (TK24928, 16,817 bp in length; Greenia 2020). To account for potential DNA degradation in the 44 museum samples, we used 4-plex reactions with dual-round enrichment and subsequent sequencing of ~3 million paired-end reads. The 12 live-trapped individuals with non-degraded DNA were enriched in a single round using a 12-plex reaction with subsequent sequencing of ~1 million paired-end reads. Enriched libraries were sequenced on the Illumina NovaSeq platform to produce paired-end reads that were 150 base pairs in length.

### Assembly and alignment

We used AdapterRemoval v.2.3.1 (Schubert et al. 2016) to trim adapters and consecutive Ns from the 5ʹ and 3ʹ ends of our sequencing reads. Quality control filters included retaining only reads with a minimum read length of 25 bp and an average base quality (Phred) score ≥20. We used Burrow-Wheeler Alignment with maximal exact matches (BWA-MEM) v. 0.7.17 (Li and Durbin 2010) to align samples to the *G. personatus* mitogenome (*G. personatus* TK24928, 16,817 bp in length; Greenia 2020). We used SAMTools v. 1.12 (Li et al. 2009) to remove duplicate and unmapped reads, and reads with an alignment quality <30, then sorted and converted the alignment files to Binary Alignment Map (BAM) format. We calculated the breadth of coverage (i.e. the percentage of the genome that had ≥1 *X*-fold read coverage) and the average depth coverage (the average *X*-fold number of reads that mapped at any location across the genome).

We then imported the BAM files into Geneious Prime v. 2022.2.1 (Biomatters, Ltd, www.geneious.com) and created a mitogenome consensus sequence that contained sites with a minimum depth of 5× reads using a majority base-call approach. In Mega v. 11.03 (Tamura et al. 2021), we manually curated our multiple-pairwise alignment by removing positions with high disagreement and low alignment quality that included portions of the D-loop and cyt-b regions. Because of the data-trimming and subsequent removal of cyt-b, we also individually aligned each read to the COX1 and cyt-b regions to a *G. bursarius* partial mitogenome (GenBank accession MZ030793.1; Greenia 2020). We focused on these regions as COX1 had the lowest adaptive/synonymous replacement (~0), whereas cyt-b is commonly used in the phylogenetic reconstruction and also has low adaptive/synonymous replacement in *Geomys* (Greenia 2020). Mitogenomes were annotated using the Basic Local Alignment Search Tool (BLAST) comparison to sequences on GenBank (Benson et al. 2013). We generated three datasets: 1) mitogenome-wide consensus sequences that were manually trimmed to remove disagreements, 2) consensus sequences based on reads that were independently aligned to the COX1 gene, and 3) consensus sequences based on reads independently aligned to the cyt-b region.

### Evolutionary substitution model, spanning networks, and genetic distances

We assessed evolutionary substitution models in MEGA v. 11.0.13 using Bayesian information criterion scores for the trimmed mitogenome-wide dataset, COX1, and cyt-b. We then generated a minimum spanning network for the mitogenome-wide dataset in POPArt v. 1.7 (Bandelt et al. 1999; Leigh and Bryant 2015), visualizing haplotype clusters based on geographic regions divided by major riverways that act as dispersal and gene flow barriers (Alexander et al. 2024), subspecies, and finally geographic distance. We also visualized median joining trees for the same three categories for the COX1 and cyt-b datasets. We only generated a minimum spanning network for the trimmed mitogenome-wide dataset as high variability and non-informative single nucleotide polymorphisms (SNPs) precluded the construction of a median-joining network. We estimated genetic distances under the K80 substitution model (Kimura 1980) using “dist.dna” in the *ape* R package (Paradis and Schleip 2019), visualizing the genetic distances with “pheatmap” in the *pheatmap* R package (Kolde 2019). Finally, we estimated haplotype diversity and nucleotide diversity for *G. b. illinoensis* for the trimmed mitogenome, COX1, and cyt-b in DnaSP6 (Rozas et al. 2017).

### Inferring phylogenetic trees

We inferred a Bayesian estimate of phylogenetic trees, using the posterior distribution as branch validation. We generated files in Beauti v1.10.4 (Drummond et al. 2012) with the Hasegawa-Kishino-Yano substitution model using a gamma-distributed rate variation allowing for invariant sites (HKY + G + I), under an uninformative constant population prior (Kingman 1982; Drummond et al. 2002). We used an unweighted pair group method with an arithmetic mean (UPGMA) tree as an initial tree and a strict clock, meaning different branches experienced the same mutation rate, which is suitable for intraspecific analyses (Drummond and Bouckaert 2015). We ran three Monte Carlo Markov Chains (MCMCs) with a 250,000,000-chain length with log outputs every 10,000 iterations. We combined the three runs using LogCombiner v.1.10.4 (Rambaut and Drummond 2018a) and assessed the combined outputs in Tracer v.1.7.2 (Rambaut et al. 2018) with a 10,000 burn-in, relying on effective sample size (ESS) and MCMC pattern. We used TreeAnnotator v.1.10.4 (Rambaut and Drummond 2018b) and FigTree v1.4.4 to quantify and visualize the phylogenetic tree topology. We also inferred tree topology using maximum likelihood in RAxML (Stamatakis 2015), for the trimmed mitogenome-wide, COX1, and cyt-b datasets but with low support, but congruent results (see Supplementary Material 2).

## Results

### Sample collection, assembly, and alignment

**Fig. 1.**
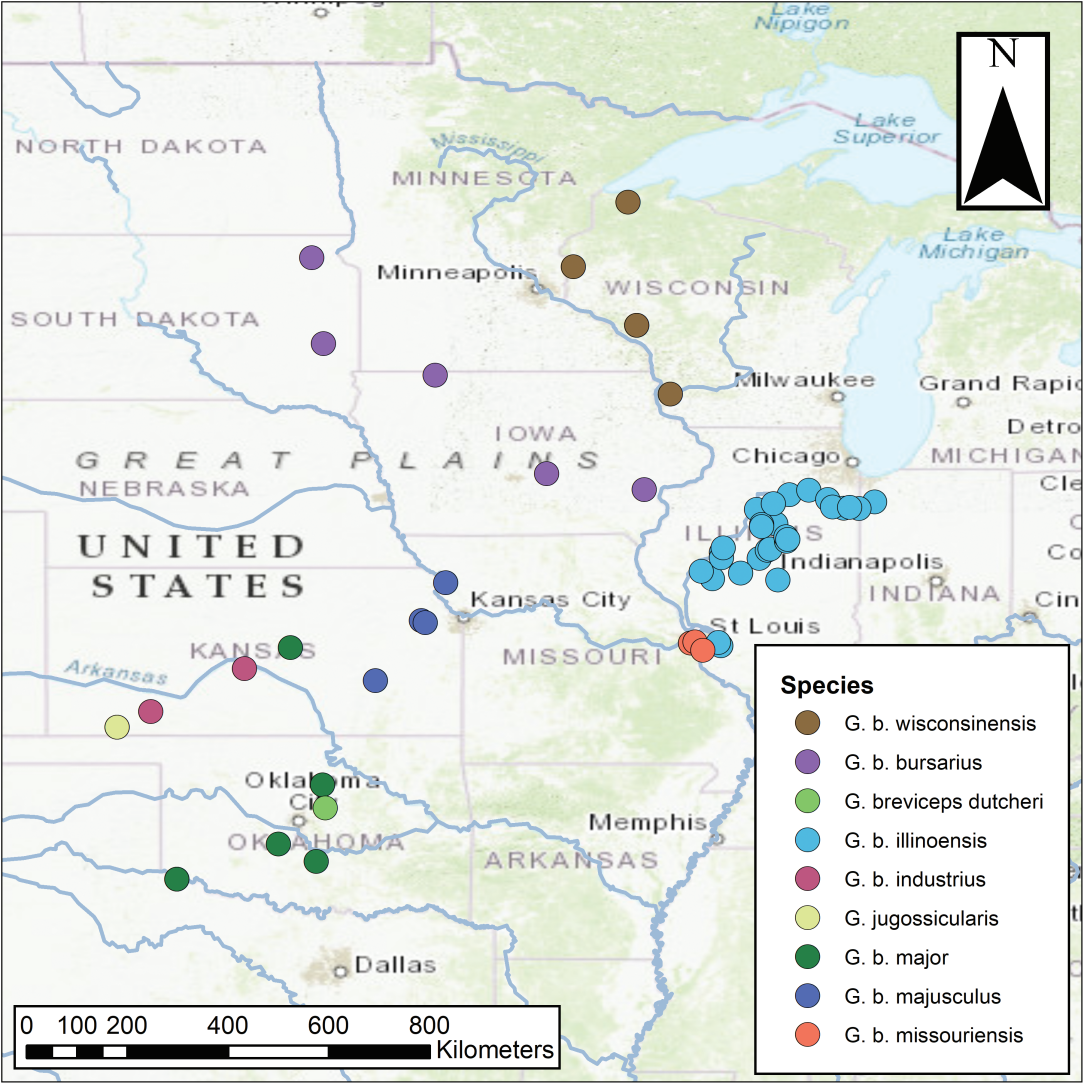
Spatial distribution of *Geomys bursarius* subspecies and two outgroups (*G. breviceps dutcheri* and *G. jugossicularis*) sampled for mitogenomes. Samples are viewed on the base World Topography Map (ESRI et al. 2017) with major rivers in the United States (ESRI 2010).

The samples from 56 individuals (Fig. 1) each had a high percentage of reads with a Phred score >30 (89.66% ± SE 0.14%; Supplementary Materials 1). The average coverage was 5,091 (SE = 94) with an average minimum coverage of 0 (SE = 0) and an average maximum coverage of 183,129 (SE = 19706). After trimming sections of cyt-b and the D-loop, we retained 14,996 bp of the mitogenome (89.17% of the reference sequence length that was used for the BWA-MEM alignment). In other *Geomys* species, the cyt-b region has been transposed into the nuclear DNA, which can co-amplify when targeting mtDNA (Nitschmann 2023), and may explain the high variation of cyt-b when aligning the full mitogenome. After aligning and trimming specifically for COX1 and cyt-b, we retained 1545 bp and 1067 bp, respectively.

### Evolutionary substitution model, spanning networks, and genetic distances

**Fig. 2.**
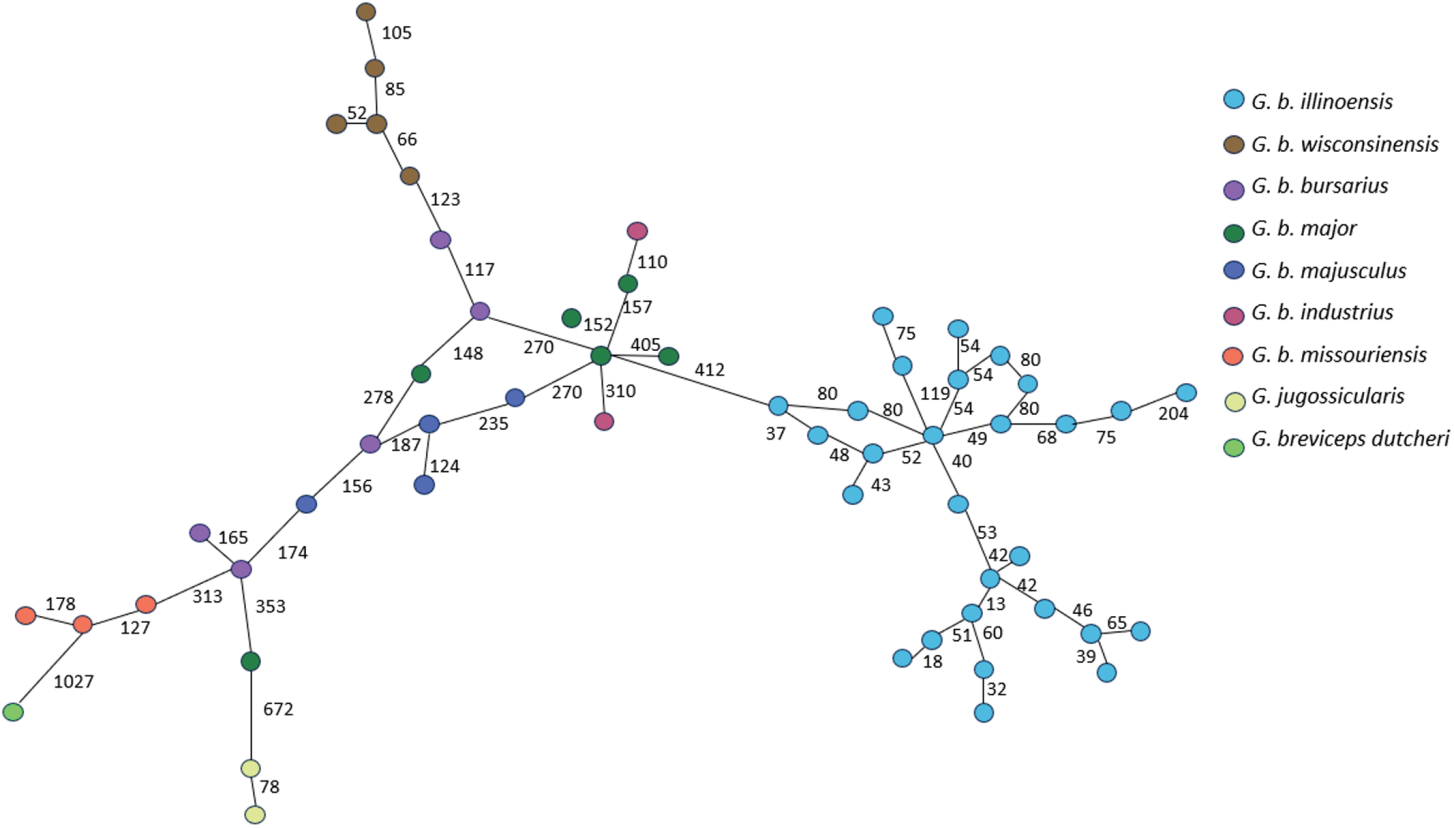
Minimum spanning tree for *Geomys bursarius* subspecies and two outgroups (*G. breviceps dutcheri* and *G. jugossicularis*) for the full mitogenomes with the number of mutations along the edges.

The best evolutionary substitution model for the mitogenome and the subregions was the HKY + G + I model, a similar model as used for *G. arenarius* (Pfau et al. 2023). From the minimum spanning tree based on 14,996 bp, *G. b. illinoensis* clustered apart from other subspecies and was separated by 412 mutations from the nearest haplotype (*G. b. major*). Although the minimum spanning tree supports the genetic separation of *G. b. illinoensis*, minimum spanning trees only denote genetic structure based on genetic distances without information about phylogeny. *Geomys bursarius missouriensis* also separated as one of the most distant clusters from *G. b. illinoensis*, indicating more genetic divergence between *G. b. missouriensis* and *G. b. illinoensis* than between other subspecies (Fig. 2; see Supplementary Material 3). *Geomys bursarius missouriensis* samples clustered with the outgroups, *G. jugossicularis* and *G. breviceps dutcheri*. *Geomys bursarsius illinoensis* as a distinct genetic unit was consistent with the median-joining trees of COX1 and cyt-b (see Supplementary Material 3). As expected, COX1 had fewer substitutions than cyt-b due to the conserved nature of the COX1 region.

**Fig. 3.**
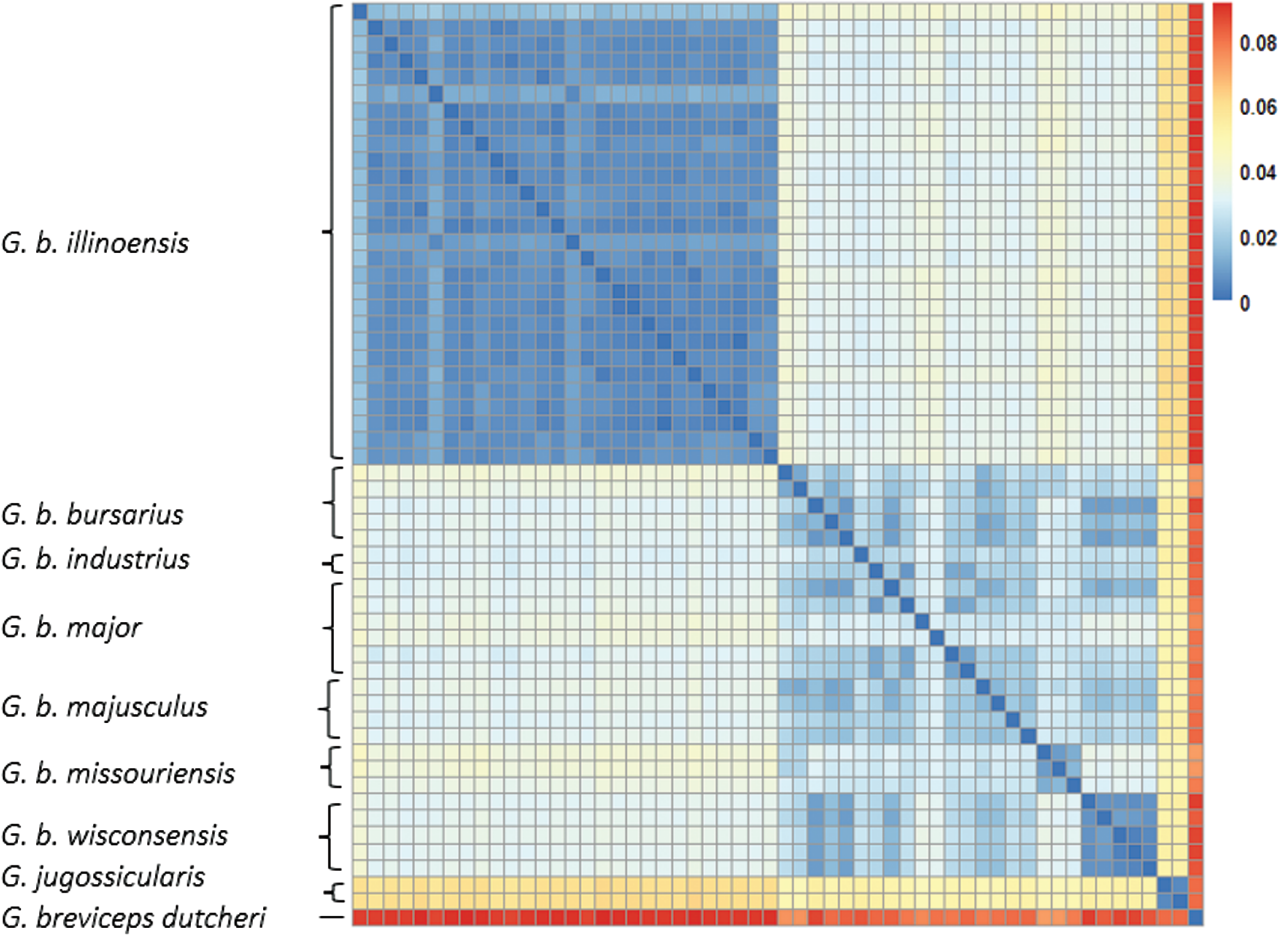
Genetic distances under the K80 substitution model for trimmed mitogenomes of *Geomys bursarius* subspecies and two outgroups (*G. jugossicularis and G. breviceps dutcheri*).

Genetic distances were lower within subspecies than between them (Fig. 3). *Geomys bursarius illinoensis*, *G. b. wisconsinensis*, and *G. b. missouriensis* had high genetic distances from the other subspecies, but *G. b. industrius*, *G. b. majusculus*, and *G. b. major* had less pronounced grouping. Across the trimmed mitogenome, there were 2,415 segregating sites and no haplotypes were the same (Table 1). When considered separately, COX1 and cyt-b had higher nucleotide diversity than the trimmed mitogenome (0.0343, 0.0461, and 0.0274, respectively). When only considering *G. b. illinoensis*, there were 562 segregating sites for the trimmed mitogenome. The nucleotide diversities of COX1 and cyt-b were higher than that of the trimmed mitogenome (0.0083, 0.0139, and 0.0071, respectively), but the COX1 diversity was only slightly higher than the trimmed mitogenome.

**Table 1.** Sample size, haplotype, haplotype diversity, number of segregating sites, number of mutations, and nucleotide diversity for the trimmed mitogenome, COX1, and cyt-b alignments for all *Geomys bursarius* subspecies, *G. jugossicularis* and *G. breviceps dutcheri* mitochondrial genomes and for only *Geomys bursarius illinoensis.*

Dataset	Sample size	Haplotypes	Haplotype diversity	Segregating sites	Mutations	Nucleotide diversity
All samples						
Trimmed mitogenome	56	56	1	2415	2625	0.0274
COX1	56	54	0.999	271	297	0.0343
cyt-b	56	56	1	239	277	0.0461
*G. b. illinoensis*						
Trimmed mitogenome	28	28	1	562	572	0.0071
COX1	28	27	0.997	61	62	0.0083
cyt-b	28	28	1	75	77	0.0139

### Phylogenetic trees

**Fig. 4.**
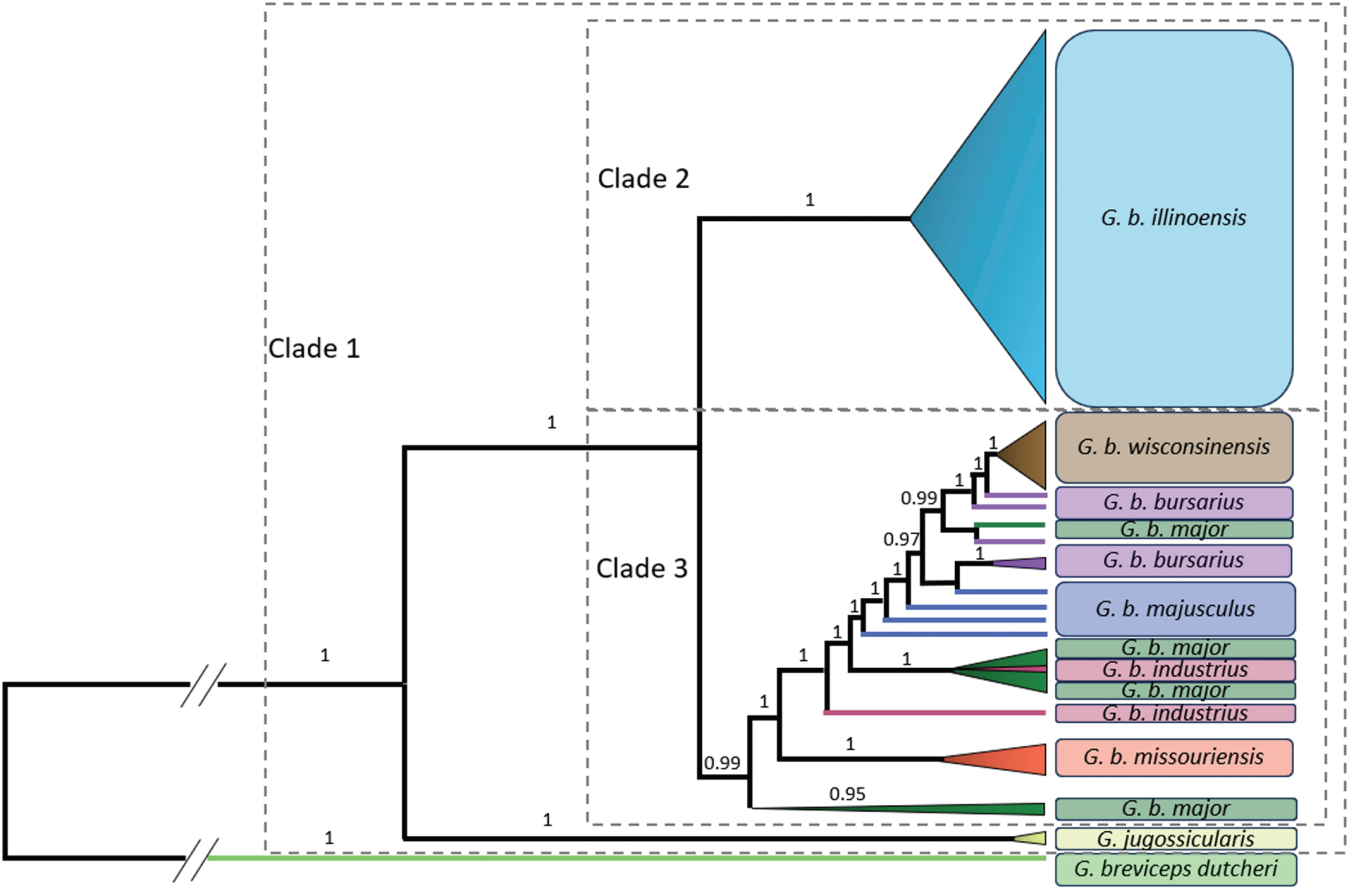
Phylogenetic tree under the HKY + G + I model in BEAST with posterior distribution support for each branch for *Geomys bursarius* and two outgroups (*G. jugossicularis* and *G. breviceps dutcheri*). Three separate clades are demarcated (dashed lines).

Bayesian methods had high support (posterior probability distributions) for phylogenetic tree topology inferred using the trimmed mitogenome-wide dataset (Fig. 4). For the Bayesian analysis, all variables had a high effective sample size (ESS) value (all ESS > 33,256), or the number of independent draws from the posterior distribution, except “meanRate,” indicating no correlation between the trees. The meanRate had a low ESS of 17 because we restricted the analysis to be a strict clock with no variation in mutation rate. Consistent with being outgroups, a midpoint-rooted Bayesian tree indicated that *G. breviceps dutcheri* is likely an outgroup to all other *Geomys* investigated in this study, *G. jugossicularis* is a sister-clade to all *G. bursarius*, and *G. b. illinoensis* forms a well resolved monophyletic clade. Although most other *G. bursarius* subspecies show geographic partitioning into distinct and well-supported clades, *G. b. major* forms a paraphyletic group with individuals clustering with *G. b. industrius* and *G. b. bursarius*, as well as a separate clade containing only *G. b. major*. BEAST identified a *G. b. industrius* sample that grouped with *G. b. major*, and this sample is likely mislabeled based on examining range maps (Schwenke 2010; Connior 2011). The maximum likelihood approach had low bootstrap support for the trimmed mitogenomes, COX1, and cyt-b, but all generated similar tree topologies (Supplementary Materials 2).

## Discussion

Multiple analyses indicate *G. b. illinoensis* experienced genetic isolation and historical divergence from the rest of the *G. bursarius* species complex. *Geomys bursarius illinoensis* forms a well-supported, monophyletic clade distinct from other subspecies. However, *G. b. illinoensis* is still within the clade that diverged from *G. jugossicularis*. These results further support the scenario of northward expansion post-glaciation with a unique colonization event across the Mississippi River. This range expansion east of the Mississippi River likely occurred farther south than the current *G. b. illinoensis* range because fossils of *Geomys* have been found south of the current distribution (Parmalee and Klippel 1981). Subsequent southern range restriction of *G. b. illinoensis* was likely due to northward expansion of deciduous forest into plains (Smith 1957; Hart 1971).

Complementary phylogenetic approaches indicate *G. b. illinoensis* is a genetically distinct population. Bayesian tree construction highly supported earlier divergence of *G. b. illinoensis* (clade 1) from the rest of the *G. bursarius* clade (clade 2; Fig. 3). The minimum spanning network, median-joining networks, and genetic distance heat map also indicate *G. b. illinoensis* is genetically differentiated. Isolation during glacial events likely increased the genetic uniqueness of this subspecies (Heaney and Timm 1983). These analyses resolve *G. bursarius* genetic relationships and expand phylogenetic understanding of a widely distributed species (Elrod et al. 2000; Sudman et al. 2006). Furthermore, our approach based on multiple, independent analyses provides a methodology that may be particularly relevant for study systems with high mitogenomic variation in local populations and that demonstrate similar ancestral dispersal followed by vicariance.

However, our phylogenetic reconstruction raises questions about other *G. bursarius* subspecies. Classification of *G*. *b. major* may need further assessment because individuals were paraphyletic and grouped with other clades as well as independently. Particularly west of the Mississippi River, hybridization at contact zones may occur and further obfuscate phylogenetic relationships (Baker et al. 1973; Tucker and Schmidly 1981; Burns et al. 1985; Dowler 1989; Genoways et al. 2008). In Kansas, where *G. jugossicularis*, *G. b. industrius*, *G. b. majusculus*, and *G. b. major* samples were collected, there is uncertain classification between *G. b. industrius* and *G. b. major* (Schwenke 2010). Furthermore, there are likely hybrid zones for *G. b. majusculus* and either *G. b. industrius* or *G. b. major* (Burns et al. 1985; Sudman et al. 1987; Schwenke 2010). Hybridization events have also been documented in Nebraska between *G. lutescens* and *G. b. majsuculus* with the Platte River acting as a barrier, possibly allowing for introgression (Genoways et al. 2008).

*Geomys bursarius major* included individuals that consistently were identified as basal clades across our phylogenetic inferences. The southernmost samples of *G. b. major* diverged the earliest within clade 3 (Fig. 3). This outcome suggests *G. b. major* was a source population or founding ancestral population from which other *Geomys bursarius* subspecies diverged. Given that some of the *G. b. major* samples came from a region that was a glacial refugia, there may be higher haplotype diversity or further divergence there than for samples closer to the *G. b. major* range limits. One *G. b. major* specimen also nested within the *G. b. bursarius* and *G. b. wisconsinensis* clade. That *G. b. major* sample was located the farthest north and may also represent a historic mitogenome retained during northward colonization and subspeciation of *G. b. bursarius* and *G. b. wisconsinensis*.

Post-glacial range expansion into the Midwest varies greatly across taxa, however, *G. bursarius* follows a phylogeographic pattern similar to other grassland species (Smith 1957; Huang et al. 2020). A grassland peninsula extended east during the Pleistocene allowing western grassland lineages to cross and establish east of the Mississippi River, including eastward dispersal of gophers (Smith 1957). However, taxa dispersed across the Mississippi River at different times (Soltis et al. 2006; Barrow et al. 2015). Some species like the chorus frog (*Pseudacris* spp.) in Illinois diverged relatively recently (Barrow et al. 2015). During the Wisconsin glaciation, an ice margin covered most of Wisconsin, but left a refugia east of the Mississippi River in the southwestern corner of the state (Clayton and Moran 1982), and a grassland peninsula covered the southern border (Tuttle et al. 2020). Given the divergence between *G. b. wisconsinensis* and *G. b. illinoensis*, *G. bursarius* likely established twice east of the Mississippi River and then was subsequently isolated in independent refugia during glaciation. Rivers including the Mississippi, Ohio, and Missouri shifted drainages during the middle Pleistocene resulting in increased flooding, channel incision, and erosion during the Holocene (Bentley et al. 2016; Fildani et al. 2018). These processes likely isolated populations that had expanded along the Pleistocene grassland peninsula (Huang et al. 2020). For grassland species like *Geomys bursarius*, forests moving northward likely further restricted populations to sandy glacial deposits, leading to isolation (Smith 1957; Barrow et al. 2015). Beyond these historic taxonomic divergence and isolating events, anthropogenic land use continues to reduce habitat suitability for relict, isolated western lineages (Barrow et al. 2015; Alexander et al. 2022), and conservation of these clades may be required to preserve unique genetic variation.

### Conservation implications

Understanding the phylogeographic history of subspecies can inform our understanding of higher taxa relationships and guide conservation goals (Avise et al. 2016; Rissler 2016). As genetic resolution increases and speciation is viewed as a continuous process, however, the identification of conservation units requires further deliberation (Coates et al. 2018). We provided evidence that *G. b. illinoensis* is phylogenetically distinct and most distantly diverged from the other *Geomys* subspecies, supporting previous work using nuclear DNA (microsatellites) that *G. b. illinoensis* is a unique population (Alexander et al. 2024). Conservation units under the Endangered Species Act often require historic divergence in both mtDNA and nuclear DNA (Avise et al. 1987; Moritz 1994, 2002; Coates et al. 2018), and *G. b. illinoensis* meets these criteria. Although analysis of complete nuclear genomes may further resolve these taxonomic relationships, analysis of nuclear microsatellites supports our findings by showing that *G. b. illinoensis* was partitioned as a separate group from subspecies west of the Mississippi River (Alexander et al. 2024).

Because *G. b. illinoensis* has experienced increased habitat fragmentation and decreased niche breadth within its range (Alexander et al. 2022), we recommend additional monitoring and land management to promote the conservation of *G. b. illinoensis*. Given that remnant groups of *G. b. illinoensis* often occur along roadway rights-of-way, roadside management that retains grassland vegetation could increase population connectivity, especially by linking areas with sandy soils (Alexander et al. 2022). Although roadsides may be suitable habitat, this may depend on the type of road (i.e. paved, gravel, or dirt), and right-of-way management along dirt roads may particularly benefit fossorial rodents (Brock and Kelt 2004). However, roads themselves may function as permeable barriers to subterranean rodents (Esperandio et al. 2019). Road underpasses, particularly as *G. b. illinoensis* is fossorial, may be a promising tool to promote connectivity, but species-specific studies are needed to determine efficacy (D’Amico et al. 2015).

Although we focus on one western lineage of gophers east of the Mississippi River, multiple grassland species in Illinois face conservation threats due to land conversion (Smith 1957; Warner 1994; Barrow et al. 2015), Thus, habitat restoration and providing connectivity between relict prairies and other grasslands should benefit gene flow for multiple species. Furthermore, taxa in Illinois may originate from clades west of the Mississippi River (Smith 1957; Barrow et al. 2015; Williams and Ibrahim 2023), so clarifying whether these eastern populations qualify as evolutionarily significant units can aid in prioritizing management actions. Understanding phylogeographic colonization events contextualizes the evolutionary trajectories and genetic structures in these unique subspecies and populations.

## Supplementary material

Supplementary material is available at *Journal of Heredity* Journal online

esae035_suppl_Supplementary_Materials

## Data Availability

BAM file Sequence Read Archive accessions SAMN41528531-SAMN41528586. Cyt-b GenBank accessions PP848353 to PP848408. Partial mitogenome, COX1, and cyt-b nexus files and Supplementary Materials 1. Dryad accession doi: 10.5061/dryad.jdfn2z3jv.
