# Supplementary material for: Phylogenetic assessment within a species complex of a subterranean rodent (Geomys bursarius) with conservation implications for isolated subspecies
Source: J Hered. 2024 Jul 10;115(5):565–74. doi: 10.1093/jhered/esae035 (PMC11334213; doi:10.1093/jhered/esae035)
Supplement: esae035_suppl_Supplementary_Materials [file esae035_suppl_supplementary_materials.zip › 2024_6_10_Alexander_gophermtDNA_Supplemental.docx]

Running Title: Gopher phylogeography using mitogenomics

Title: Phylogenetic assessment within a species complex of a subterranean rodent (*Geomys bursarius*) with conservation implications for isolated subspecies

Nathan Alexander^1^, Alida de Flamingh^2^, Bradley J. Cosentino^3^, and Robert L. Schooley^1^

^1^Department of Natural Resources and Environmental Sciences, University of Illinois, 1102 S Goodwin Ave, Urbana, IL 61801

^2^Carl R Woese Institute for Genomic Biology, University of Illinois, 1206 W Gregory Dr, Urbana, IL 61801

^3^Department of Biology, Hobart and William Smith Colleges, 300 Pulteney St, Geneva, NY 14456

Corresponding author: Nathan Alexander (nba2@illinois.edu)

Nathan Alexander ORCID: 0000-0001-9714-4761

Alida de Flamingh ORCID: 0000-0003-1223-6654

Bradley J. Cosentino ORCID: 0000-0002-6740-3133

Robert L. Schooley ORCID: 0000-0001-5951-2340

**Supplementary Materials 1:** File name, specimen catalogue identification, species identification, sex, longitude, latitude, state, county, sampling location from catalogue tag, collection year, collection month, collector, SRA accession number, GenBank accession for cyt-b, BAM file names, index, total passing reads, percent of passing reads > Q30, and read coverage min, max, and mean.

[see spreadsheet Sample_and_file_information.csv]

**Supplementary Material 2** Maximum likelihood analysis using 10,000 bootstraps under the HKY 85 model. We ran RAxML for the trimmed mitogenome dataset, COX1, and cyt-b. Bootstrap values were used to describe topology support, and trees were visualized in FigTree v1.4.4**.** For Maximum Likelihood tree inference, we rooted trees based on the midpoint with bootstrap values for branch support (Fig. A.1). There was poor bootstrap support and phylogenetic resolution of other *Geomys* subspecies, likely due to low sample size (not enough informative sites) and high genetic variation (low-frequency variants that obfuscate topologies).


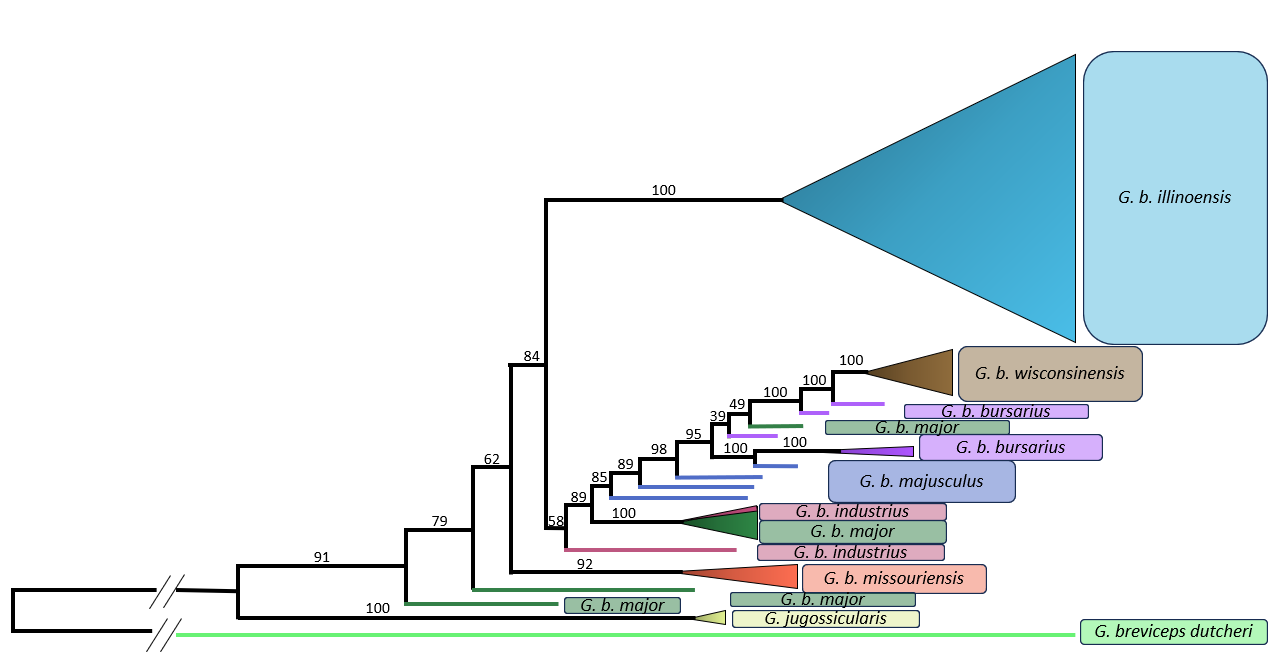


**Fig. S2.1**  RAxML phylogenetic tree of gophers using the trimmed mitogenome with bootstrap values (n = 10,000) along branches under the HKY 85 model.


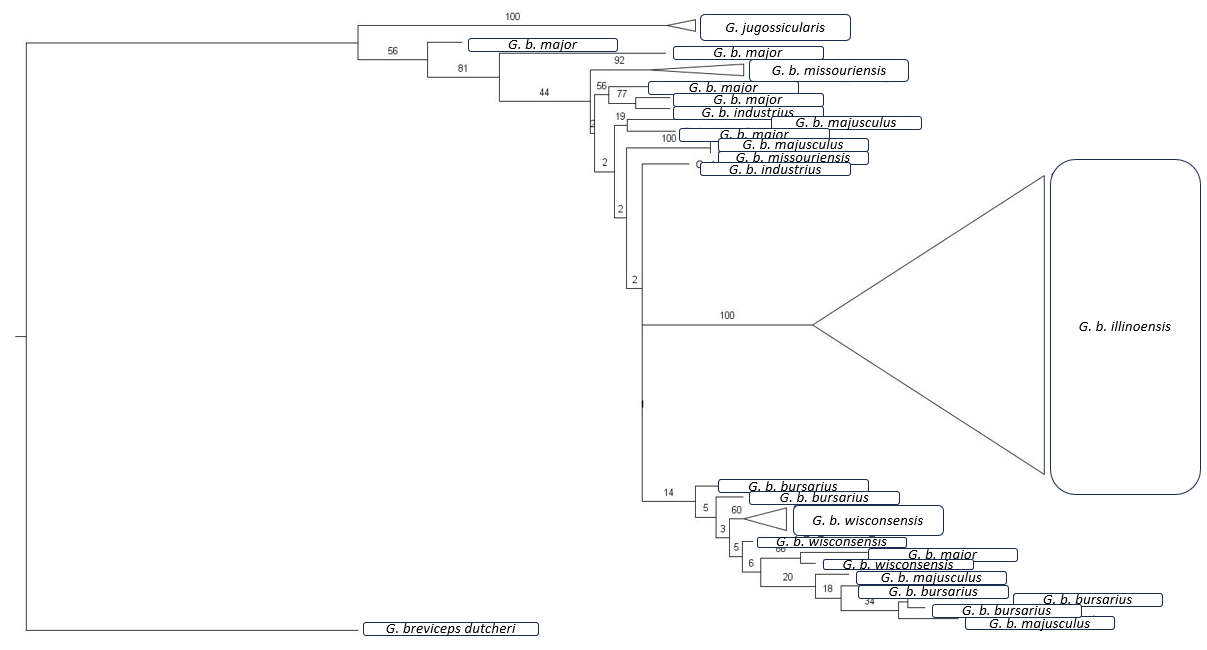


**Fig. S2.2**  RAxML phylogenetic tree of gophers using the COX1 mitogenome region with bootstrap values (n = 10,000) along branches under the HKY 85 model.


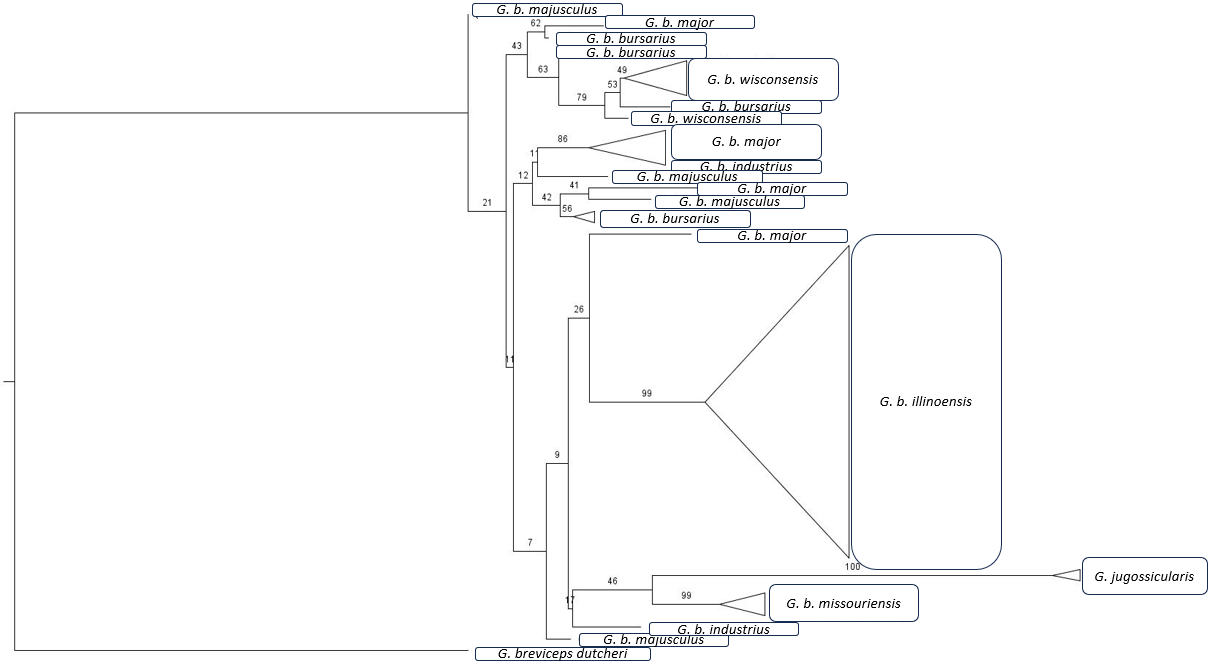


**Fig. S2.3**  RAxML phylogenetic tree of gophers using the cyt-b mitogenome region with bootstrap values (n = 10,000) along branches under the HKY 85 model.

**Supplementary Material 3**. Minimum spanning network of the retained mitogenome and the median spanning networks for COX1 and cyt-b


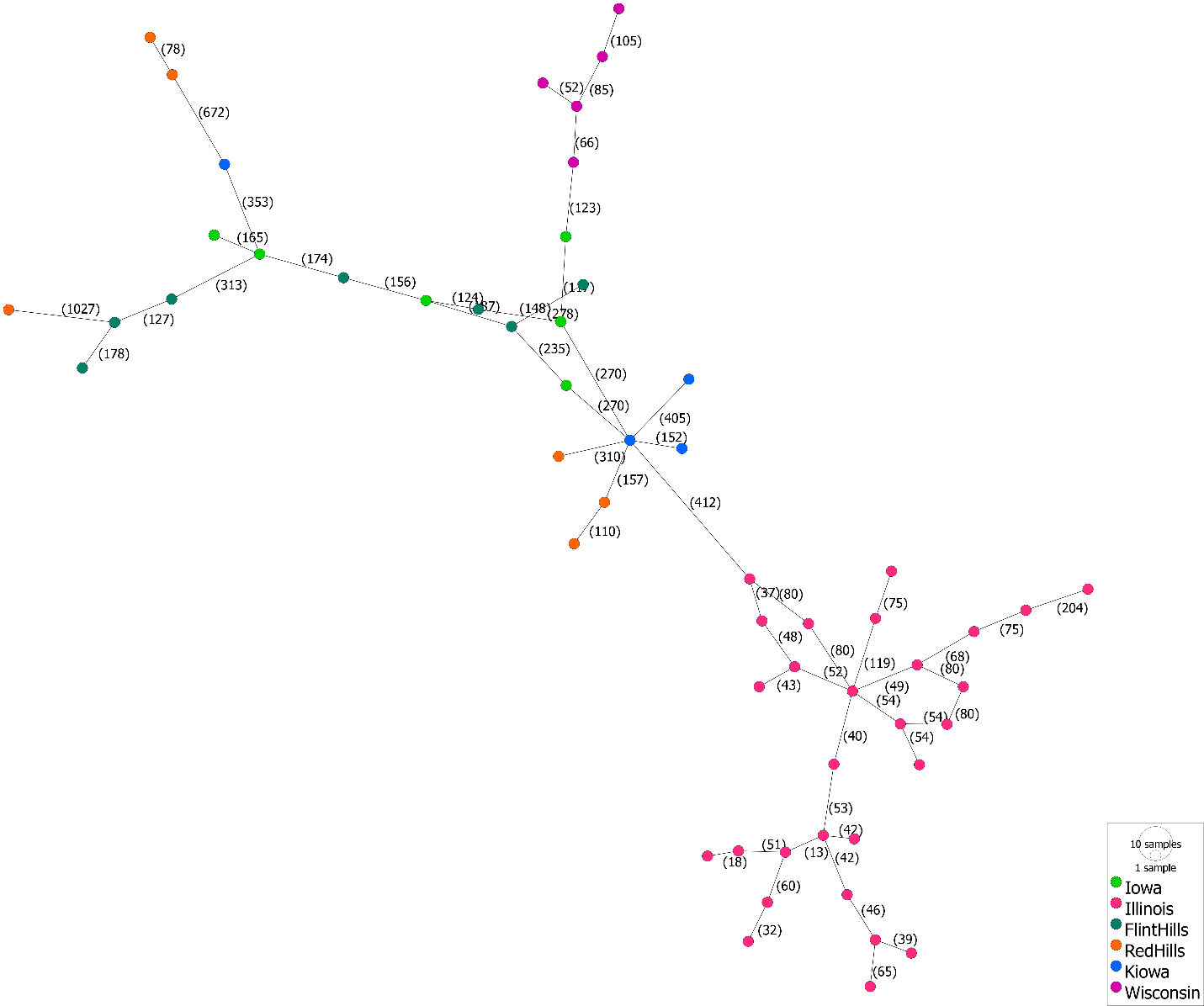


**Fig. S3.1** Minimum spanning tree based on regions (Alexander et al. 2024) for the trimmed mitogenomes.


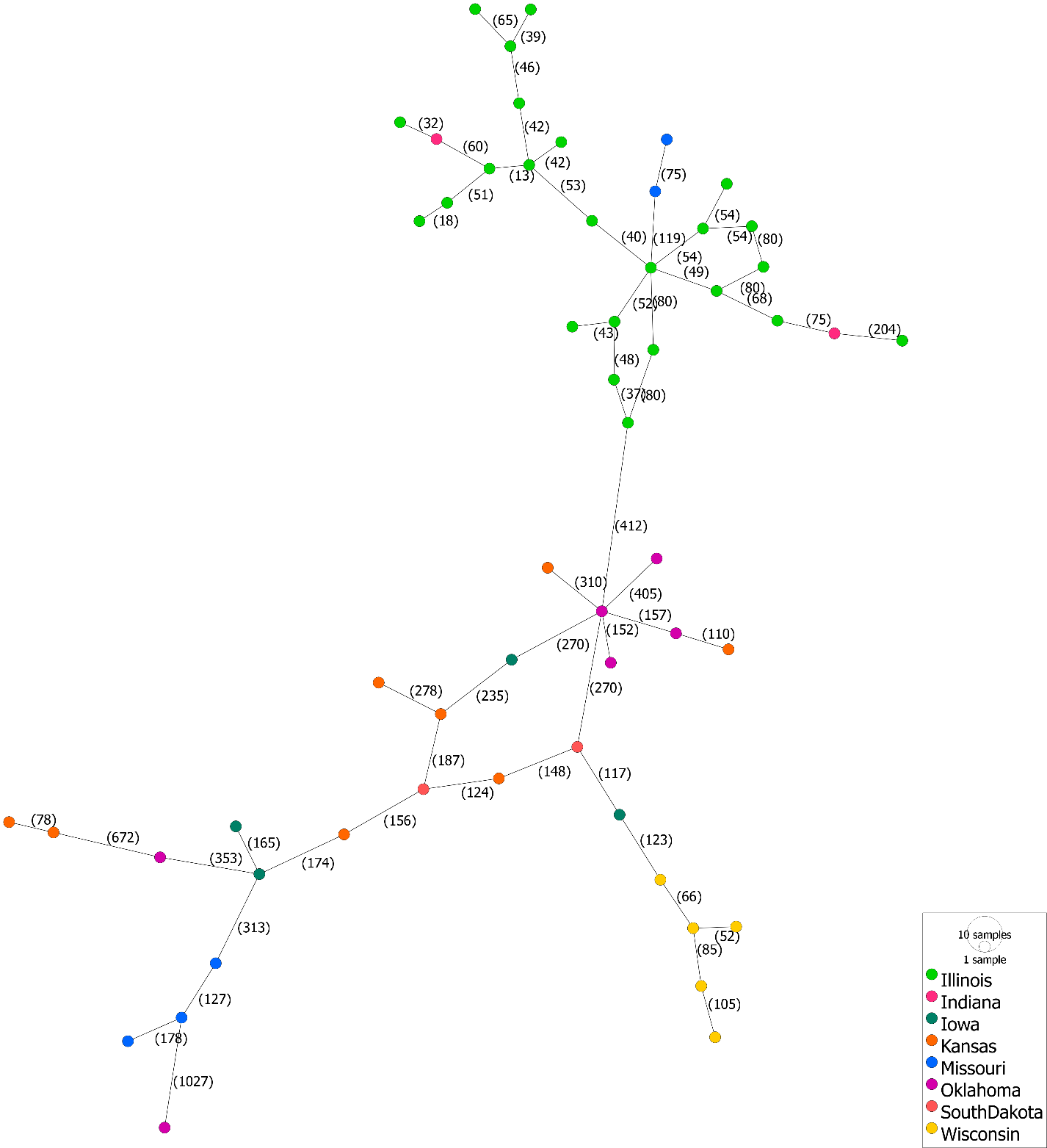


**Fig. S3.2** Minimum spanning tree using geographic distance identified to states for the trimmed mitogenome.


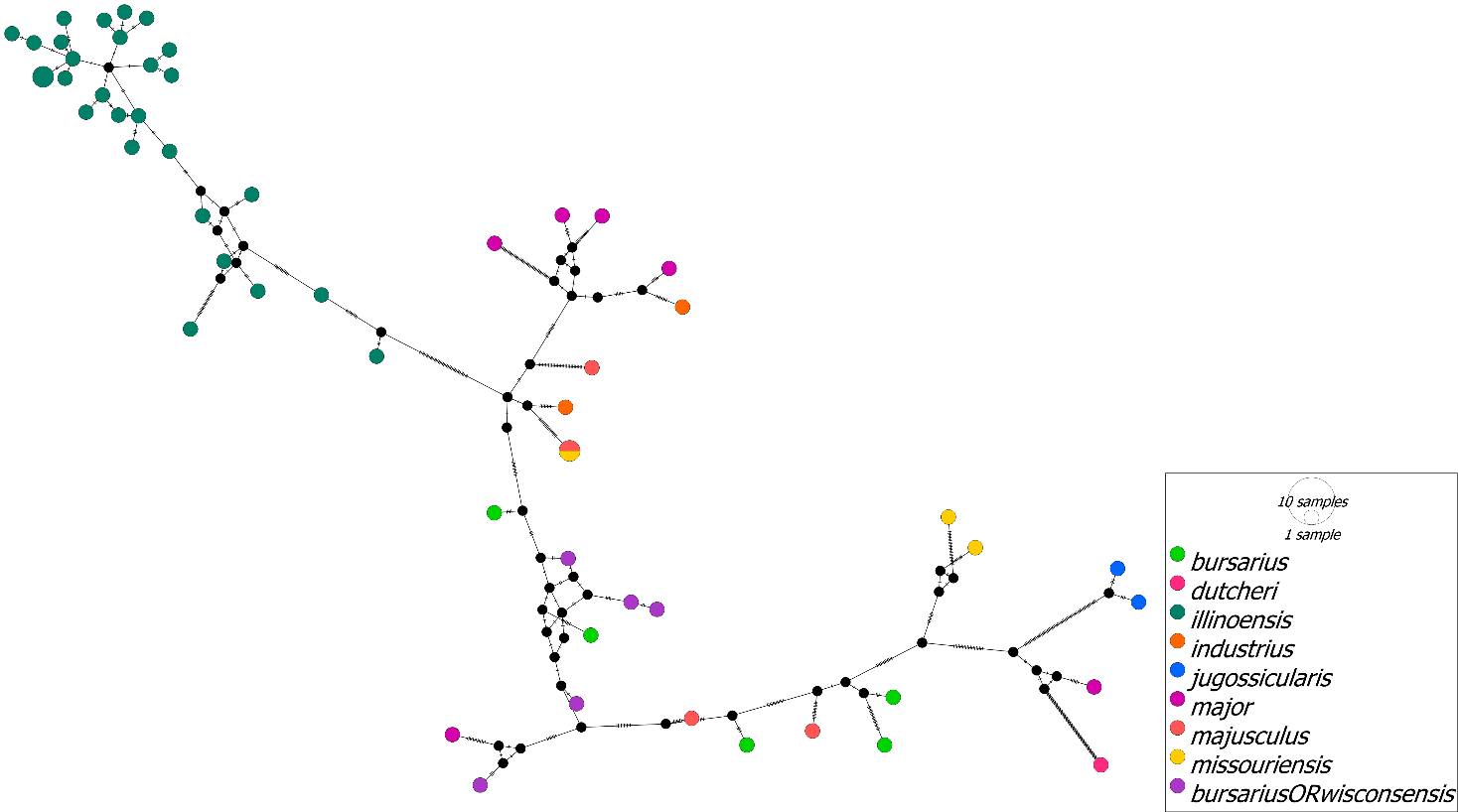


**Fig. S3.3** Median joining network by subspecies for COX1 (bp = 1545) from PopArt. Epsilon = 0.


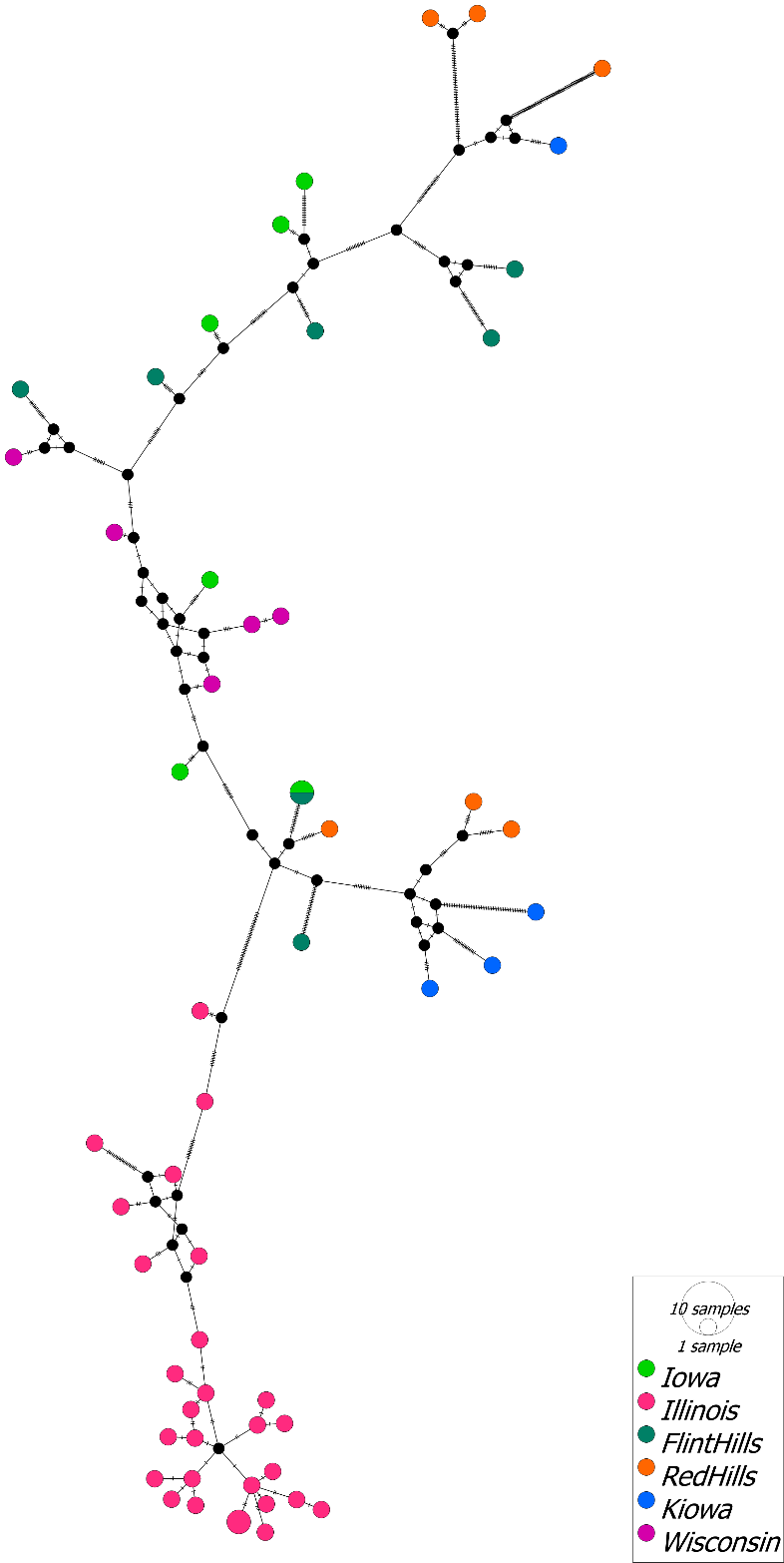


**Fig. S3.4** Median joining network by region for COX1 (bp = 1545). Epsilon=0.


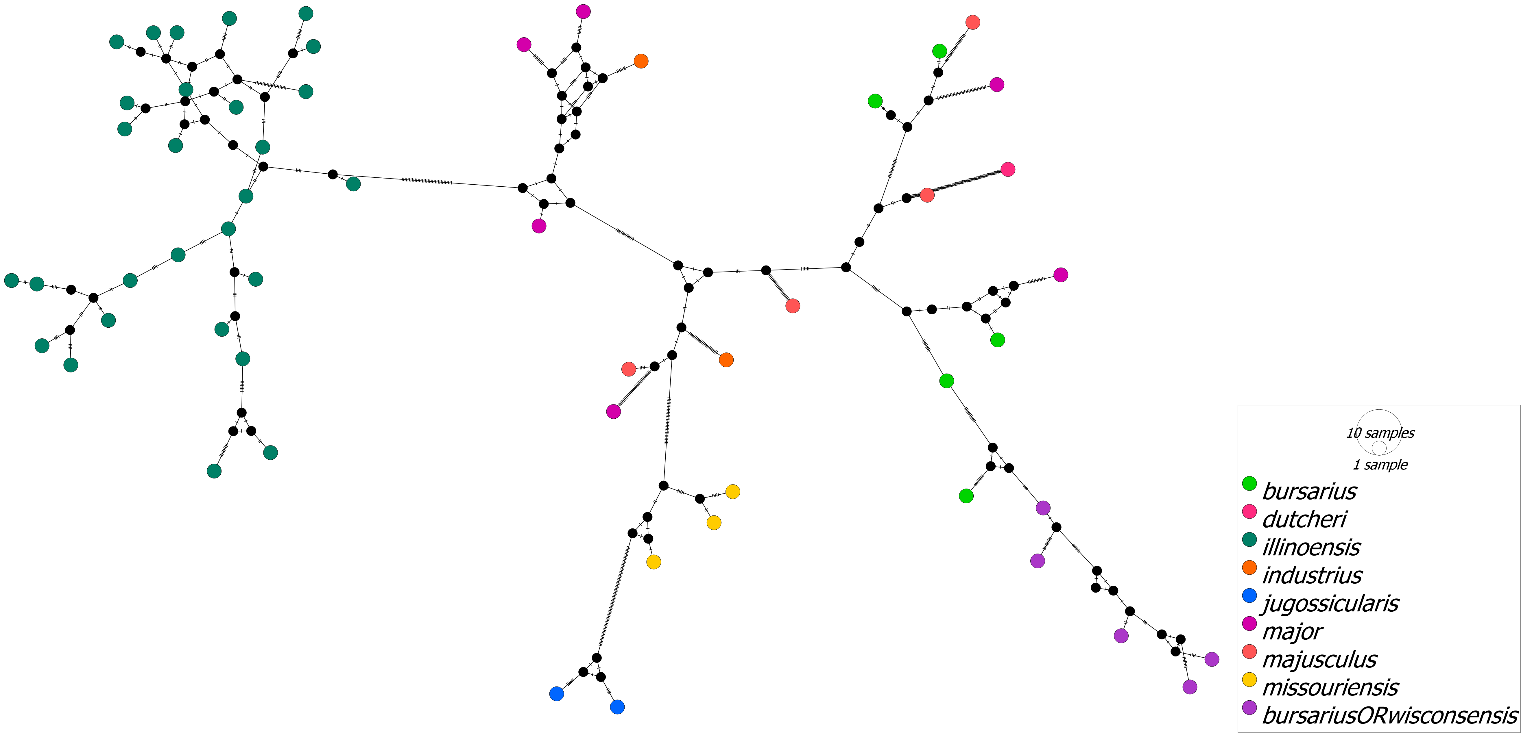


**Fig. S3.5** Median joining network for cyt-b (1067 bp) by species. PopArt, epsilon=0.


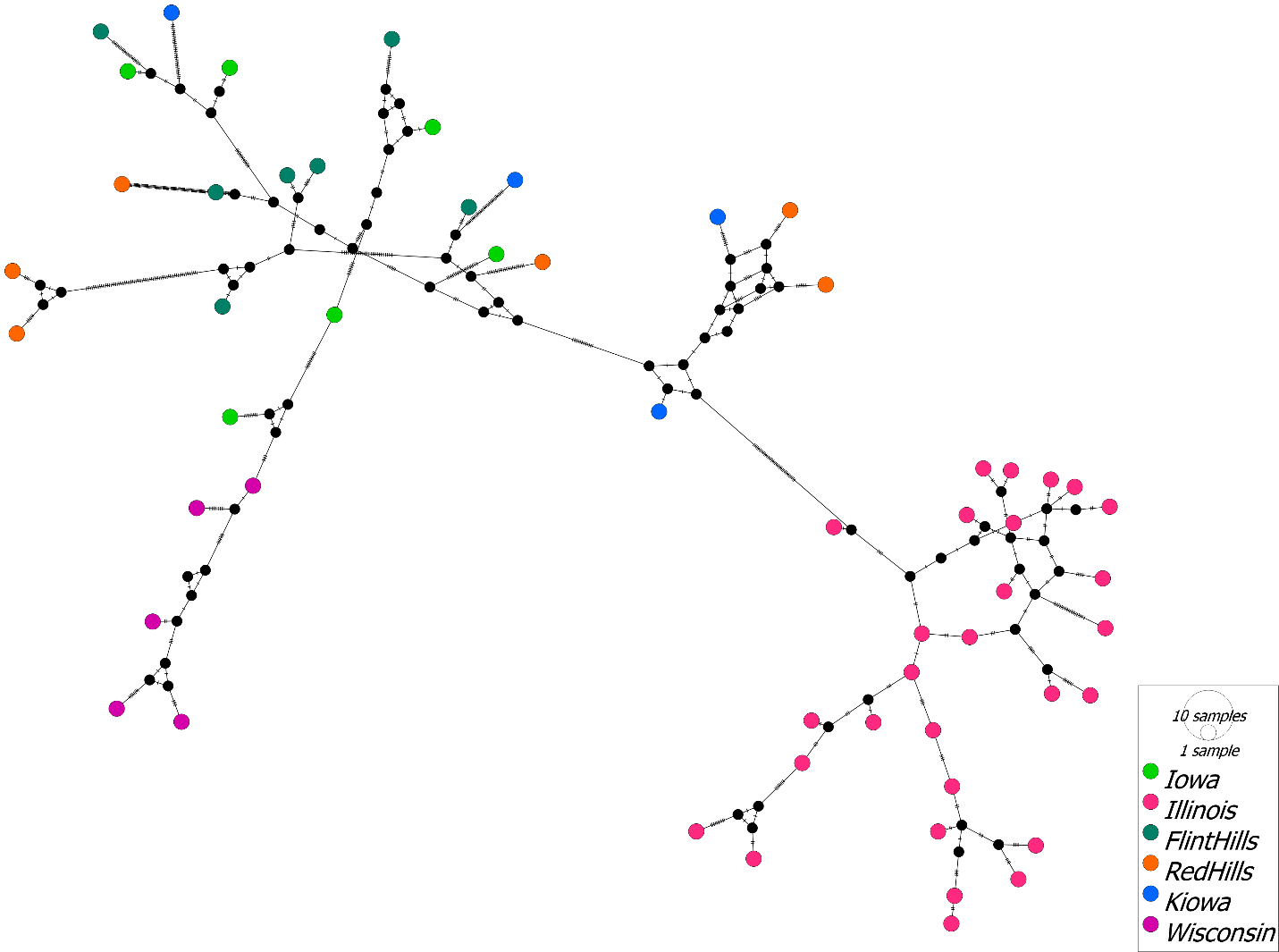


**Fig. S3.6** Median joining network for cyt-b (bp=1067) by region (Alexander et al. 2024). PopArt, epsilon=0.

**Literature Cited**

Alexander, N., B. J. Cosentino, and R. L. Schooley. 2024. Partitioning genetic structure of a subterranean rodent at multiple spatial scales: accounting for isolation by barriers, distance, and environment. Landscape Ecology 39:92.
